# Psychometric Properties of the Persian Version of the Center for Epidemiological Studies Depression Scale Among the Iranian Public People During COVID-19 Pandemic

**DOI:** 10.3389/fpubh.2021.728904

**Published:** 2021-12-14

**Authors:** Hamid Sharif Nia, Pardis Rahmatpour, Erika Sivarajan Froelicher, Saeed Pahlevan Sharif, Omolhoda Kaveh, Azadeh Rezazadeh Fazeli, Chin Chin Sia

**Affiliations:** ^1^Traditional and Complementary Medicine Research Center, Addiction Institute, Mazandaran University of Medical Sciences, Sari, Iran; ^2^Department of Nursing, Alborz University of Medical Sciences, Karaj, Iran; ^3^School of Medicine, University of California, San Francisco, San Francisco, CA, United States; ^4^Faculty of Business and Law, Taylor's University, Subang Jaya, Malaysia; ^5^Department of Nursing, School of Nursing and Midwifery, Mazandaran University of Medical Sciences, Sari, Iran; ^6^Department of Psychology, Islamic Azad Univeristy, Tonekabon, Iran

**Keywords:** depression, COVID-19, Iran, validation, reliability

## Abstract

**Background:** Several studies indicate a high prevalence of depression around the world during the period of the COVID-19 pandemic. Using a valid instrument to capture the depression of an individual in this situation is both important and timely. The present study aims to evaluate the psychometric properties of the Persian version of the Center for Epidemiological Studies Depression Scale (CES-D) among the public during the COVID-19 pandemic in Iran.

**Method:** This is a cross-sectional study that was conducted in the Iranian population (*n* = 600) from April to July 2020. A two-part online form was used: sociodemographic characteristics and depression items (CES-D). The construct validity and internal consistency reliability of the scale were evaluated.

**Result:** The results of the exploratory factor analysis illustrated two factors with 43.35% of the total variance of the depression were explained. Confirmatory factor analysis indicated that this model fits well. Internal consistency reliability was evaluated, and it was acceptable.

**Conclusion:** The findings demonstrated that, in the Iranian sample, this depression scale yielded two factors (somatic and positive affects) solutions with suitable psychometric properties.

## Introduction

The COVID-19 pandemic has caused major world health concerns. The emergent global challenge that began in Wuhan city, China, led to high hospitalization and mortality rates (1). Unfortunately, due to its very high transmission rate, the virus spread rapidly throughout the world and infected almost all countries worldwide in a short time (2).

The implementation of quarantine measures, such as home quarantines and lockdowns, to deal with the pandemic has affected the normal life of a person (3, 4). In other words, as well as the public physical health concerns and human safety, this disease has caused several mental health problems and psychological symptoms (5–7), resulting in social and economic consequences during the COVID-19 outbreak (8). These consequences are significant and may have long-lasting effects (3). Wang et al. reported in their study that 53.8% of the general Chinese population experienced psychological consequences during the outbreak (1). A variety of symptoms were experienced, such as despair, fear of prolonged quarantine, the uncertainty of the future, fear of illness, boredom, misinformation, inadequacy, Post-Traumatic Stress Disorder (PTSD) (9), confusion, anger, depression, anxiety, stigmatization, avoidance behaviors, increased smoking, and alcohol consumption, and have been reported in several studies (5, 10–14). Due to its prevalence and associated consequences, depression is an important health problem. With a worldwide prevalence of about 10–15%, it is one of the most frequent and debilitating mental disorders (15). The results of qualitative research of Iranian students showed that these people had unique experiences that have created negative emotions, such as confusion, feeling downbeat, exhibiting obsessive behaviors, fear of the future, and worries about the family's economy and family health and welfare (3).

The current treatments on COVID-19 worldwide have chiefly concentrated on the implementation of strategies for infection control, identification and treatment of patients, and reduction of death (16). The psychosocial aspect of the COVID-19 pandemic has yet to be thoroughly considered (17). One of the most important and popular psychological consequences is depression, which has been reported in various studies (18, 19). Gao et al. study reported 48.3% depression symptoms among Chinese citizens (20). Liu et al. showed depression symptoms were 53.5% in the general public in China (6). Also, a systematic review and meta-analysis that included 14 studies in the Iranian general population during the COVID-19 outbreak demonstrated that the depression prevalence was 33.7% (21). The results of these studies documented the high prevalence of depression around the world, thus requiring special attention.

Numerous scales were used to study depression during the COVID-19 pandemic in the general population [e.g., self-rating depression scale (SDS) (22, 23), Hospital Anxiety and Depression Scale (HADS) (24), Depression, Anxiety and Stress Scale (DASS-21) (1, 18, 25), Patient Health Questionnaire-9 (PHQ-9) (19, 26, 27), Goldberg Depression and Anxiety Scale (GAD-7) (28), The Center for Epidemiological Studies Depression Scale (CES-D-20) (28) and Online Ecological Recognition (OER) (19)]. Among the various tools, CES-D appears to be an acceptable tool for assessing and screening individuals with depressive symptoms in the general population (29).

The CES-D was developed by Radloff (30). It is a tool widely used in population research to assess four dimensions of mood and includes positive mood (four items; e.g., being hopeful or feeling happy), physical symptoms (seven items; e.g., changes in appetite, sleep disorders, or difficulty walking), depressed mood (seven items; e.g., feeling upset, lonely, sad, and crying), and interpersonal relationships (two items; e.g., the feeling that others do not like me or that they are unfriendly) (30). It has been validated in different countries with various groups: in a population of patients with systemic lupus erythematous in Canada (31), Chinese primary care samples (32), American psychiatric samples (33), in a French adolescents sample (34), and patients with cancer in Persia (35). The results of a systematic review study showed that most of the studies provided support from the four-factor structure consistent with the original scale. The majority of these studies were conducted in the United States. Versus, studies conducted in Asia, reported two or three factors. This finding indicates differences in a participant cohort and context and that culture and ethnicity have a significant influence on the structure of factors (36).

The psychometric properties of the Persian version of the CES-D have been evaluated and were confirmed in patients with cancer in Iran (35); therefore, it was used in the present study. Given the alarming rise in depression in Iran during the current crisis and the prevailing context of the Iranian society, the present study was performed to evaluate the validity and reliability of the Persian version of the CES-D among a general population during the COVID-19 pandemic in Iran. It is both timely and important that a valid and reliable scale be identified to capture the depression of an individual in this situation.

## Methods

### Study Design

A methodological and cross-sectional design was used to answer the research question. The study was conducted in an Iranian population between April and July 2020. Ethics approval of this study was obtained from the Mazandaran University of Medical Sciences Research Ethics Committee (IR.MAZUMS.REC.1400.10526).

### Sample

#### Describe the Sample and Sampling Technique

The inclusion criteria for participation were all adults (>18 years old) who were willing to participate in this study. The sample size for performing factor analysis is between 5 and 10 samples per item of the scale (37). In this study, 600 Iranian adults were recruited into the study *via* a random online data gathering. The total questionnaire was prepared using Google form and was sent to the Iranian Telegram public groups. Finally, a sample of 300 was used for Exploratory Factor Analysis (EFA), and another sample of 300 was used to evaluate Confirmatory Factor Analysis (CFA).

### Measures

The questionnaire was composed of sociodemographic information and the Persian version of the CES-D that was translated by Sharif Nia et al. (35). This scale is used to measure depression-related symptoms experienced over a week. A four-point Likert scale, ranging from 0 = rarely or never (<1 day); 1 = occasionally or in few times (1–2 days); 2 = occasionally or a moderate amount of time (3–4 days); and 3 = most of the time or all the time (5–7 days). The construct validity of the CES-D (16 items) is defined of three factors: positive affect (four items), negative affect (five items), and somatic affect (seven items) (35).

### Data Analysis

The construct validity of scales was assessed using Maximum Likelihood Exploratory Factor Analysis (MLEFA) with Promax rotation. The Kaiser–Meyer–Olkin test (KMO) and Bartlett's test of sphericity were used to evaluate the study sample. The factor extraction was based on absolute factor loading values that should be >0.3, Eigenvalues >1, communalities >0.2, and scree plots (38). Confirmatory Factor Analysis (CFA) was estimated using the most common model fit indices, such as Root Mean Square of Error of Approximation (RMSEA), Comparative Fit Index (CFI), Parsimonious Normed Fit Index (PNFI), Parsimonious Comparative Fit Index (PCFI), Tucker–Lewis index (TLI), Incremental Fit Index (IFI), and CMIN/DF. Items with standardized factor loading lower than 0.5 were excluded from the model.

In order to assess the reliability, internal consistency was measured by Cronbach's alpha (α), McDonald's omega (Ω), maximum reliability (Max R), and average inter-item correlation (AIC). Coefficients α and Ω values >0.7 (39) and AIC between 0.2 and 0.4 indicated good internal consistency and were acceptable (40). Also, composite reliability (CR) value >0.7 was considered fit and acceptable (41). Before conducting factor analysis, the assumption of univariate and multivariate normality and outliers were examined. All data were analyzed using SPSS-AMOS_24_, SPSS R-Menu2, and JASP_0.13.1.0_.

## Results

In this study, the psychometric properties of the Persian version of the Center for Epidemiological Studies Depression Scale were assessed in the general population. This scale had acceptable validity and reliability and explained 43.35% of the variance. The mean and standard deviations of age of the participants were 34.04 (±9.1) years. The majority of the samples were females (*n* = 439, 73.2%). Other demographic characteristics of the participants are shown in Table 1.

**Table 1:** Demographic characteristics of participants (*n* = 600).

**Variables**	**n (%)**
**Gender**	
Female	439 (73.2)
Male	161 (26.8)
**Marital status**	
Single	262 (43.7)
Married	338 (56.3)
**Education level**	
Less than diploma	23 (3.8)
Diploma	72 (12)
Bachelor	264 (44)
Master/Ph.D.	241 (40)
**Employment**	
Unemployed	159 (26.5)
Employed	331 (55.2)
Student	110 (18.3)
**History of COVID-19**	
Yes	144 (24)
No	456 (76)
**Family history of COVID-19**	
Yes	291 (48.5)
No	309 (51.5)

The KMO test value was 0.851, and Bartlett's test value was 1287.143 (*p* < 0.001). The two factors extracted after conducting EFA were *somatic* and *positive affects* (Table 2—these two factors explained 43.35% of the total variance of the CES-D).

**Table 2:** Exploratory factors extracted from the Persian version of CES-D (*n* = 300).

**Factors**	**Q_**n**_. Item**	**Factor loading**	**h^**2**^**	**Eigenvalue**	**% Variance**
Somatic	5. I could not get “going.”	0.719	0.517	2.80	23.35
	4. My sleep was restless.	0.698	0.412		
	8. I felt that I could not shake off the blues even with help from my family or friends.	0.638	0.410		
	6. I had trouble keeping my mind on what I was doing.	0.556	0.355		
	2. I did not feel like eating; my appetite was poor.	0.544	0.279		
	11. I had crying spells.	0.535	0.320		
	7. I talked less than usual.	0.516	0.298		
	1. I was bothered by things that usually don't bother me.	0.483	0.232		
Positive affect	16. I enjoyed life.	0.860	0.773	2.40	20.00
	15. I was happy.	0.825	0.765		
	14. I felt hopeful about the future.	0.768	0.543		
	13. I felt I was just as good as other people.	0.628	0.355		

**Figure 1:**
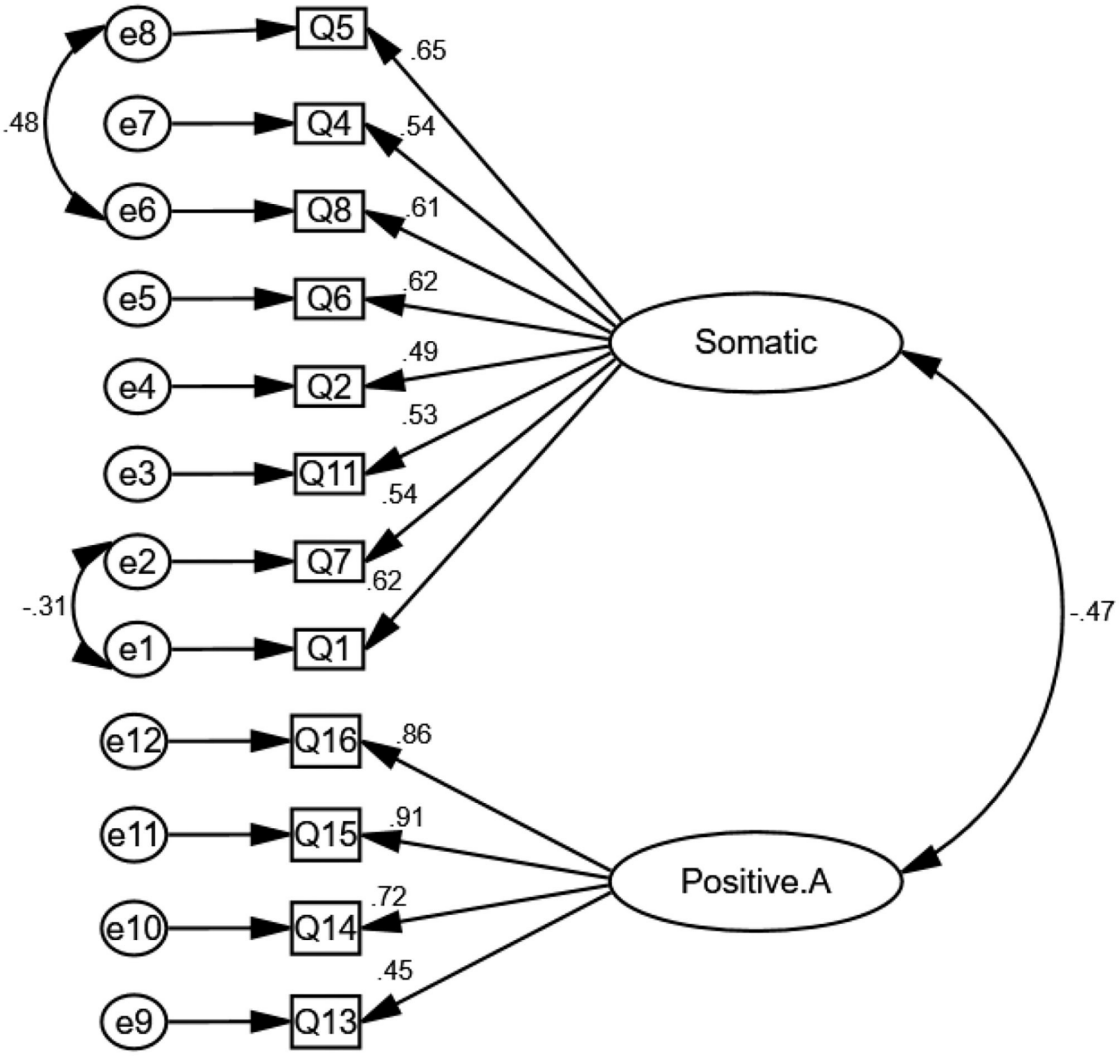
The two-structure model of the Persian version of sociodemographic characteristics and depression (CES-D) (*n* = 300).

The CFA results indicated a good model of fit; [*χ*^2^(51) = 107.040, *p* < 0.001, χ^2^/*df* = 2.09, CFI = 0.95, IFI = 0.95, TLI = 0.94, PCFI = 0.73, PNFI = 0.70, RMSEA (90% C.I.) = 0.060 (0.044, 0.077)] (see Figure 1). In addition, the internal consistency of the Persian version of CES-D scale was excellent (Table 3).

**Table 3:** The indices of the reliability and internal consistency of the Persian version of CES-D.

	**CR**	**MaxR**	**Alpha (CI 95%)**	**Omega**	**AIC**
Somatic	0.80	0.83	0.80 (0.76–0.83)	0.83	0.34
Positive affect	0.85	0.89	0.85 (0.82–0.87)	0.88	0.59

## Discussion

The present study evaluated the reliability and validity of the Persian version of the CES-D in the population during the COVID-19 pandemic in Iran. The various ethnic groups may have different factor structures (42), so it was important to test whether the scale is valid and reliable for this population during the COVID-19 pandemic. The findings of our study demonstrated a two-factor structure for the CES-D scale: somatic symptoms (eight items) and positive affect (four items). Our two-factor structure accounted for 43.35% of the total item variance. In social science studies, we are confronted with subjective and attitudinal concepts. It is recommended to consider a solution that accounts for 60 percent of the total variance (43). The total extracted variance may indicate that the data are not useful and may need to revisit measures and even the data collection process. So, there are most likely chances of more factors showing up than the expected factors in a model.

These results are similar to those found by Dam and Earleywine in a general population, Thanh et al. in adolescents, and Kwakkenbos et al. in patients with systemic sclerosis who all identified that the CES-D scale includes two factors (positive affect and negative affect) (44–46). Also, Adams et al. reported two latent factors [diminished positive affect (DPA) and interpersonal negative affect (INA)] that emerged from the scale among black men (47).

Other researchers have identified three factors, suggesting that the differences of those studies and the ones that identified two factors were in the type of factors; for example, a three-factor structure of the CES-D, reported in a sample of Arabic females, including positive affect and interpersonal problems, somatic symptoms (48). Also Sharif Nia et al., in Iranian patients with cancer, found three-factor consisting of somatic affect, negative affect, and positive affect (35). A three-factor structure, consisting of interpersonal problems, positive affect, and a combination of somatic symptoms and a depressive mood, was reported in many studies (49–52). On the other hand, several studies have reported four-factor models of the CES-D scale (32, 44, 53–55).

The first factor identified in this study was somatic affect with eight items. These items were related to walking, sleeping, energy, mindfulness, appetite, crying, talking, happiness, and enjoyment. According to the literature, somatic affect can relate to change in appetite, fatigue, lack of energy, sleep disturbance, pain and general aches, and concomitant organic medical conditions (e.g., headache, backache, and arthritis) (56, 57). The occurrence of somatic complaints as one of the symptoms of depression has been proved to subsequently relate to life-threatening diseases like cardiovascular disease, stroke, hypertension, diabetes, and low health-related quality of life that eventually led to death (58). Also, the patients with major somatic affect had recurrent periods of depression with greater severity as well as further depressive symptoms rather than the patients without somatic affect (59). Finally, the presence of these symptoms imposes a large economic burden on the family and society (60). This finding was confirmed in previous studies (32, 35, 49, 51, 52, 54).

Another factor identified in the present study was positive affect. It consists of four items. Blanco and Joormann showed positive affect was related to depression (61). This is an important adaptive role to benefit health and improve treatment outcomes (62). Moreover, Ahadi et al. found that positive affect could reduce the progression of depression (63). A positive affect makes individuals more resilient to negative life situations (64). Accordingly, a diminished positive affect can lead to depression and its adverse consequences (65). For the reasons mentioned above, depression is an important issue at this time of the COVID-19 pandemic.

## Strengths and Limitations of This Study

Filling the gap of limitation of valid and reliable scale, in general, Persian language community, acceptable sample size according to the COSMIN checklist and assessing the Composite Reliability and Omega coefficient are the strength of this study. The first limitation for our study is recall bias because the data were collected by self-reported questionnaires online. In addition, the use of a convenience sample may result in a sample that is not entirely representative of the population of Iran. The majority of the participants were female based on the public Iranian population *via* an online questionnaire; therefore, the gender balance was not possible.

## Implications

The Persian version of the CES-D can be administered by health care providers, such as nurses, psychologists, and psychiatrists, to screen for symptoms of depression among the population during the COVID-19 pandemic to identify people at high risk and ultimately prevent the progression of depression, which may cause irreversible complications.

## Conclusion

According to the results of the present study, the Persian version of the CES-D scale had acceptable construct validity and reliability. It identified two factors with 12 items that explained 43.35% of the total variance of depression of the Iranian population during COVID-19. This scale can be useful for researchers and psychologists to assess depression during the COVID-19 pandemic.

## Data Availability Statement

The data that support the findings of this study are available from the corresponding author upon reasonable request.

## Ethics Statement

The studies involving human participants were reviewed and approved by Mazandaran University of Medical Sciences Research Ethics Committee (IR.MAZUMS..REC.1400.10526). The patients/participants provided their written informed consent to participate in this study.

## Author Contributions

Material preparation and data collection performed by OK, PR, and AR. SP confirm this edit and HS analyzed data. The first draft of the manuscript was written by OK, ES, and CS. All authors contributed to the study conception and design, commented on previous versions of the manuscript, and read and approved the final manuscript.

## Conflict of Interest

The authors declare that the research was conducted in the absence of any commercial or financial relationships that could be construed as a potential conflict of interest.

## Publisher's Note

All claims expressed in this article are solely those of the authors and do not necessarily represent those of their affiliated organizations, or those of the publisher, the editors and the reviewers. Any product that may be evaluated in this article, or claim that may be made by its manufacturer, is not guaranteed or endorsed by the publisher.
